# Lifestyles of *Gypsy*-family transposons shape their regulatory mechanisms

**DOI:** 10.1073/pnas.2617602123

**Published:** 2026-08-12

**Authors:** Anna-Maria Papameletiou, Benjamin Czech Nicholson, Susanne Bornelöv, Gregory J. Hannon

**Affiliations:** ^a^ Cancer Research UK Cambridge Institute, University of Cambridge, Cambridge CB2 0RE, United Kingdom; ^b^ Department of Biochemistry, University of Cambridge, Cambridge CB2 1GA, United Kingdom

**Keywords:** transposable elements, *Drosophila*, transcriptional regulation

## Abstract

Transposable elements (TEs) are a highly diverse group of selfish genomic elements, prevalent across the tree of life, whose uncontrolled propagation poses a threat to genome stability. Recent studies have explored the evolution of *Drosophila melanogaster* TEs, their coevolution with the host genome, and mechanisms that regulate their activity. However, little is known about their cross-species evolutionary patterns. Long terminal repeat (LTR) retrotransposons are the most active group of TEs in *Drosophila*. They are broadly separated into retroelements, which are active in the germline, and insect endogenous retroviruses that express in the gonadal soma. Somatic elements are hypothesized to infect the germline through their acquisition of virus-derived proteins such as Envelope and sORF2, thus multiplying through successive generations. In this study, we curated the sequences of LTR retrotransposons in 249 drosophilid genomes, allowing us to study their evolution across these species and highlight their varying degrees of conservation. Furthermore, we reveal multiple instances of Envelope protein loss or inactivation that suggest shifts in the expression pattern of these transposons, likely accompanied by adopting different transcriptional control mechanisms. We contrast this with the evolutionary history of sORF2, which we found to be much more stable. Lastly, we examine variations in transposon LTR regions responsible for transcriptional regulation and use predictive modeling to identify six transcription factors that may regulate their tissue-specific expression. Altogether, we reveal complex, interspecies evolutionary patterns of *Gypsy*-family LTR retrotransposons and highlight examples of their coevolution with their host genome.

## Significance

Transposable elements (TEs) are “selfish” genomic elements that, in addition to vertical inheritance from parent to offspring, can be shared between species and thereby become established in new hosts. To better understand the scale of this phenomenon, we analyzed 53 TEs across 249 fly species. Our analyses point toward a high degree of similarities in the TE landscape between distantly related species and identified putative transfer events in the TE evolutionary histories. Furthermore, we identified conserved strategies that TEs use to mediate their transcriptional activity. These results offer insights into the genomic arms race which drives host-parasite coevolution.

Transposable elements (TEs) are mobile genetic elements that threaten to compromise genomic integrity. TEs constitute roughly 20% of the *Drosophila melanogaster* genome, 45% of the human genome, with much higher burden observed in other eukaryotes such as salamanders and some plants (1, 2). The interplay between TEs and host genomes is complex. TEs propagate by eluding host silencing mechanisms and, consequentially, the host adapts to counteract those challenges. This can be described as an evolutionary arms race and this ongoing molecular battle drives evolution, resulting in the rapid diversification of TE-related sequences both within and across species.

TEs are categorized according to their transposition mechanism (3). Of particular interest are *Gypsy*-family long terminal repeat (LTR) retrotransposons that move via a “copy-and-paste” mechanism and are the most prominent elements in the *D. melanogaster* genome (4). These TEs are further subdivided according to their evolutionary relationships and include *Gypsy/gypsy (Metaviridae)*, *Gypsy/mdg1*, *Gypsy/mdg3,* *Gypsy*/*chimpo*, and *Gypsy/osvaldo* (5–7).

All *Gypsy-*family LTR retrotransposons contain two identical LTRs surrounding an internal region containing open reading frames (ORFs) for Group-specific Antigen (*GAG*), which encodes structural proteins, and Polymerase (*POL*), which contains activities facilitating reverse transcription and integration (8). An interesting characteristic of the *Metaviridae Gypsy/gypsy* clade is the acquisition of cell-to-cell movement capabilities, which splits the clade into retroelements and insect endogenous retroviruses (ERV). The latter contain an additional ORF encoding Envelope (ENV), which enables soma to germline infectivity by *gypsy* (9–12) and other TEs from the *Gypsy/gypsy* clade of which *gypsy* is a member (13, 14). The Envelope protein shares evolutionary origin with *Baculoviruses* (15–17) and is thought to only have been acquired once by an ancestral *Drosophila* transposon (7, 18).

In ERVs, the F-type glycoprotein allows the formation of virus-like particles (VLPs), allowing the spread of ERV content among different cell types via endocytosis (7, 19, 20). More recently, sORF2, a fusion associated small transmembrane (FAST)-like protein, has been implicated in soma-to-germline transfer of all four *Gypsy/mdg1-*family transposons (21). FAST proteins facilitate viral spread by mediating membrane fusion, enabling cell-to-cell transfer without an extracellular phase (22). sORF2, in particular, has been shown to form channels between cells to facilitate transfer of TE material (21).

TEs, like other genomic content, can be vertically transmitted from one generation to the next. However, their evolutionary histories are often much more complex due to introgression (23) which is widespread in the *Drosophila* evolutionary history (24) and horizontal transposon transfer (HTT). HTT is common in insects, including members of the *Drosophila* clade, where up to 55% of the total genome may have arisen as an indirect result of HTT (25). HTT has been proposed to occur via direct transfer through a VLP (20), piggybacking on exogenous vectors such as viruses (26) and parasitic mites and wasps (27, 28). Additionally, parasitic bacteria called *Wolbachia* have been observed to horizontally transfer genes among *Drosophila* species (29), and similar transfer of TEs has been hypothesized (30). Both introgression and HTT enables TEs to invade new species: In total, out of 122 well-characterized *D. melanogaster* TEs, an estimated 11 have invaded the species in the past 200 y (25). The majority of these come from species within the *Sophophora* subgenus, *D. simulans,* and *D. willistoni* (31); however, the more distant *Zaprionus* genus—which branched from the *D. immigrans* subgroup and is now nested within the paraphyletic *Drosophila* genus—also has an extensive history of HTT with the *D. melanogaster* clade (18, 32–34).

To counteract potentially deleterious effects of TEs on the host genome, various host defense mechanisms have evolved to silence these elements. In *Drosophila*, this is mainly driven by the PIWI-interacting RNA (piRNA) pathway, a small-RNA based system that mediates TE silencing at both the cotranscriptional and posttranscriptional levels (35, 36). piRNAs against newly invaded TEs are thought to arise when the transposon inserts antisense into a piRNA cluster (18, 37). Conversely, TEs carry their own tissue-specific regulatory identity: The LTR regions contain transcription factor binding sites (TFBS) which enable transcriptional activity specifically in the cell types in which the elements are active (38–42).

Recently, there has been a burst in the availability of *Drosophila* genome assemblies due, in part, to the more widespread availability of long read sequencing technologies (43, 44). In this study, we took advantage of these large resources and annotated *Gypsy*-family TEs in 248 non–*D. melanogaster* drosophilid species. Our LTR-specific pipeline allowed us to capture and categorize more TEs than had been seen by previous methods. Using this resource of curated TEs, we focused on the proteins associated with cell-to-cell infectivity, ENV and sORF2. We show that sORF2 is highly conserved across drosophilids, whereas ENV is repeatedly lost. Lastly, we identified TFBS motifs associated with *ENV* presence and absence, allowing us to hypothesize that TEs hijack ovarian development pathways to ensure their transcriptional activation via host-intrinsic pathways. Altogether, our work provides detailed insights into the lifestyle of *Gypsy* TEs in drosophilids.

## Results

### Most *Gypsy* TEs Are Present Beyond the *Melanogaster* Clade.

The *D. melanogaster* genome contains thousands of TE insertions (3) representing over 120 distinct TE families, each defined by its own consensus sequence. A subset of these, belonging to the *Gypsy* family, are further divided into *Gypsy/gypsy*, some of which contain the *env* gene, *Gypsy/mdg1*, which harbor *sORF2*, and others (*mdg3, *osvaldo*, chimpo*) (7). To understand the evolutionary origin of *ENV* and *sORF2* and their role in defining TE lifestyles, we analyzed 450 publicly available, long-read genome assemblies (44) representing 313 different species and spanning 10 genera: *Amiota, Chymomyza, Caconexus, Drosophila, Hirtodrosophila, Lordiphosa, Scaptodrosophila, Scaptomyza, Zaprionus,* and *Zygothrica*. We excluded 201 assemblies due to redundancy or lower quality (*SI Appendix*, Fig. S1*A*), leaving 249 species with a high-quality genome. Together, this collection spans approximately 50 My of evolution (24)—with the lowest distance between species being 0.6 My (between *D. cyrtoloma* and *D. melanocephala*) (45, 46)—as well as diverse geographic spread (*SI Appendix*, Fig. S1 *B* and *C*).

**Fig. 1.**
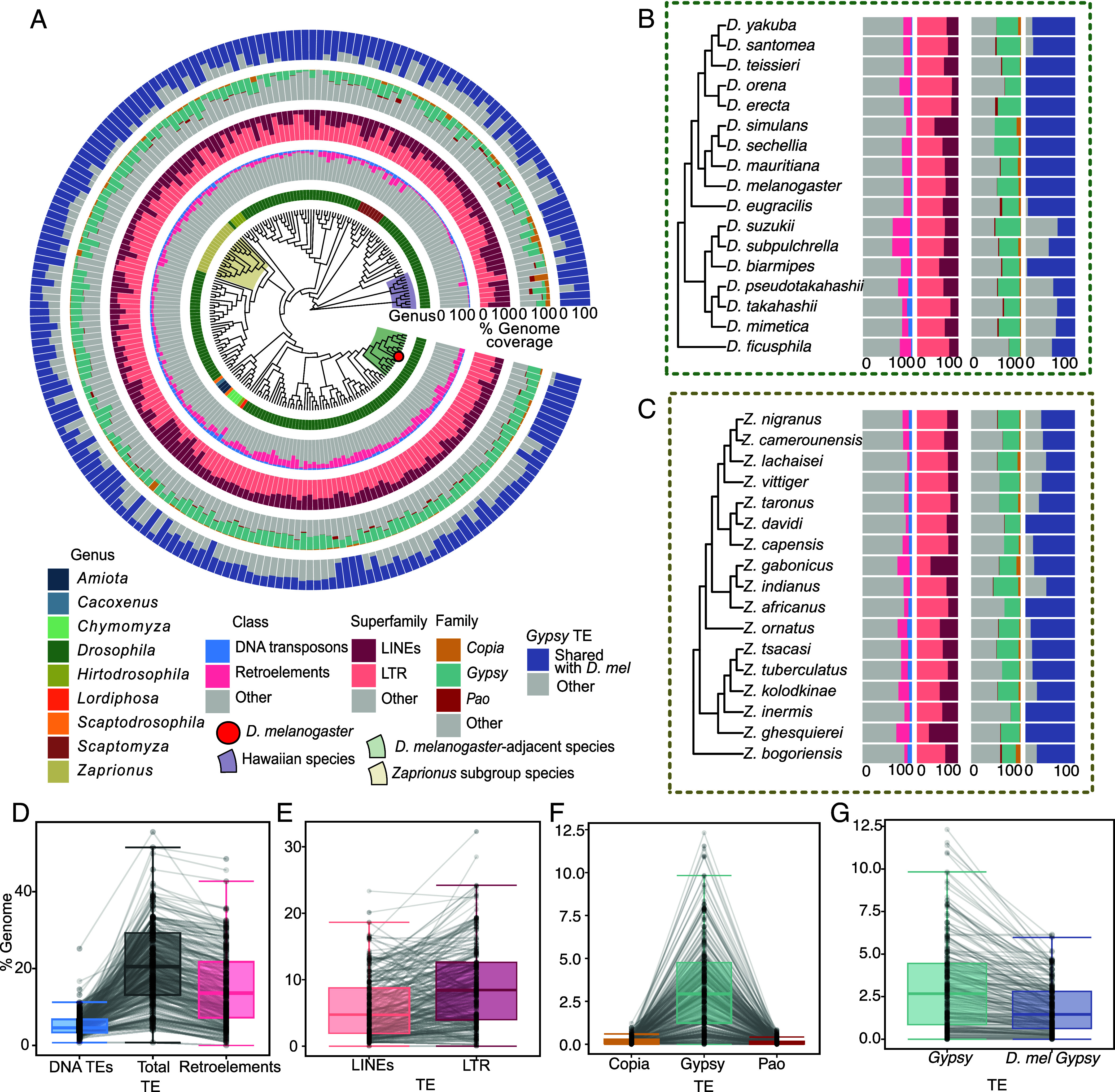
An overview of TE profiles in 249 drosophilid species. (*A*) Phylogenetic tree of 249 drosophilid species. Genus is indicated by the innermost circle, followed by the percentages of TE class, retrotransposon superfamily, and LTR family for each species as well as the percentage of *D. mel*-like *Gypsy*-family TEs, obtained by EDTA and RepeatMasker. Shaded regions on the tree highlight the *melanogaster* clade (green; *D melanogaster* shown as a red circle) and the *Zaprionus* genus (yellow), which are also shown in panel *B* and *C*. (*B* and *C*) Phylogenetic trees show a zoom-in on the information shown in (*A*) for the *D. melanogaster* clade (*B*) and the *Zaprionus* genus (*C*). (*D*–*G*) Paired box plots show the percentage of the genome covered by TEs, grouped by TE class (*D*), retrotransposon superfamily (*E*) and LTR family (*F*) and *D. mel*-like *Gypsy-*family TEs (*G*) for each species. Individual points between box plots are joined when they represent values from the same species.

We assessed the repeat landscapes of the 249 genomes using the Extensive de novo TE Annotator (EDTA) (47). Overall, we detected transposon content up to 55.5% (*Lordiphosa clarofinis*) of the total genome size (Dataset S1). As expected, larger genomes were characterized by higher TE proportions (Pearson’s R = 0.94, *P* < 2.2 × 10^−16^) (*SI Appendix*, Fig. S2*A*), which may in part reflect more complete genome assemblies. Notable exceptions were the *Amiota*, *Chymomyza,* and *Caconexus* genera, characterized by a lower-than-expected retroelement content, while their DNA transposon content followed the same trend as other species (*SI Appendix*, Fig. S2 *B*–*D*). These three genera therefore have a different TE landscape compared with other drosophilids. An overview of genomic TE content across all 249 genomes is shown in Fig. 1*A*. Overall, retroelements occupied a median 2.7-fold greater fraction of the genomes as compared with DNA transposons (Wilcoxon signed-rank test, *P* < 2.2 × 10^−16^) (Fig. 1 *A* and *D* and *SI Appendix*, Fig. S3). Within the retroelements, LTR retrotransposons occupied a median 1.8-fold greater fraction than LINE elements (Wilcoxon signed-rank test, *P* < 2.2 × 10^−16^) (Fig. 1 *A* and *E* and *SI Appendix*, Fig. S3). Lastly, among LTR retrotransposons, the *Gypsy* family has the highest genomic coverage (Fig. 1 *A* and *F* and *SI Appendix*, Fig. S3), exceeding that of both *Copia* (median 25-fold lower, Wilcoxon signed-rank test, *P* < 2.2 × 10^−16^) and *Pao* (median fivefold lower, Wilcoxon signed-rank test, *P* < 2.2 × 10^−16^). The *D. melanogaster* clade (i.e., 10 species most closely related to *D. melanogaster*), ranging from *D. yakuba to D. melanogaster* (Fig. 1*B*), all showed similar TE profiles, however, there were several differences in more distantly related species, for instance with higher overall TE content (39.68%) in *D. suzukii.* Lastly, since *Gypsy*-family TEs covered the largest fraction of drosophilid genomes, we asked how similar these were to the *D. melanogaster* reference TEs. To quantify this, we searched using RepeatMasker (48) for sequences resembling the *D. melanogaster Gypsy*-family reference TEs (hereafter referred to *D. mel*-like) in each of the 248 genomes as well as the *D. mel* genome. Notably, we found species with up to 100% of their *Gypsy* TE content being *D. mel*-like (Fig. 1 *A* and *G* and *SI Appendix*, Fig. S3). Species closely related to *D. melanogaster* (Fig. 1*B*), in the *Zaprionus* (Fig. 1*C*) genus, and a few other species evolutionarily distant to *D. melanogaster* had the highest proportions of *D. mel*-like *Gypsy* TEs. We concluded that *D. melanogaster*’s TE content, measured in genomic coverage, is highly consistent across the *melanogaster* clade (Fig. 1 *A* and *B* and *SI Appendix*, Fig. S3) and also comparable to *D. melanogaster* in more evolutionarily distant species, albeit with variation across different clades (Fig. 1 *A*–*G* and *SI Appendix*, Fig. S3).

### A Pipeline to Annotate *Gypsy*-Family LTR Retrotransposons in Drosophilids.

**Fig. 2.**
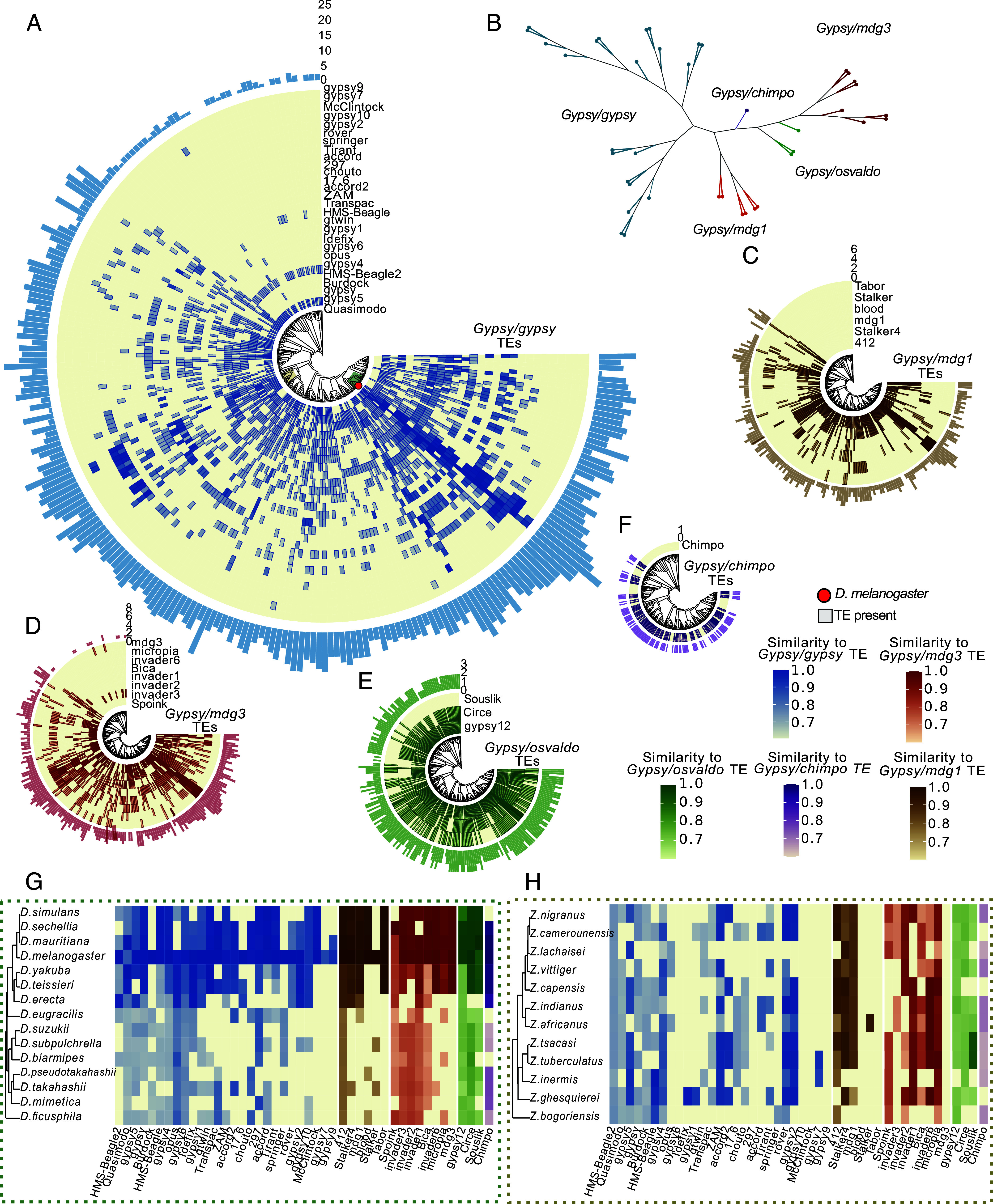
The presence of *Gypsy*-family LTR retrotransposons. (*A*) A phylogenetic tree and accompanying heatmap show *Gypsy/*gypsy TE presence (≥70% similarity) and absence across 249 species. Heatmap colors indicate the similarity to the corresponding *POL* ORF in *D. melanogaster.* The bar plot represents the total number of *D. melanogaster Gypsy/*gypsy TEs identified in each species. (*B*) Displayed is an unrooted phylogenetic tree of the D. *melanogaster Gypsy*-TE *POL* consensus sequences, colored by the TE clade to which they belong. All internal nodes have a bootstrap support of ≥67%. (*C*–*F*) Same as (*A*), but showing *Gypsy/mdg1* TEs (*C*), *Gypsy/mdg3* TEs (*D*), *Gypsy/osvaldo* TEs and *Gypsy/chimpo* TEs (*F*). (*G* and *H*) Phylogenetic trees show in detail the same information as (*A* and *C*–*F*) for the *D. melanogaster* clade (*G*) and the *Zaprionus* genus (*H*).

*Gypsy*-family TEs are the most diverse and prominent class in *D. melanogaster*. Having established similarities between the TE contents of *D. melanogaster* and other drosophilids, we focused on identifying which *D. melanogaster*-like TEs were present in the other species. This is challenging, as prevalent HTT combined with rapid evolution makes ortholog assignment across TEs particularly difficult. To this end, we used hidden Markov models (HMM) to identify *Gypsy*-family TEs in drosophilid genomes. We started with previously constructed and partially curated TE libraries (18) from which we filtered for *Gypsy*-family transposons and extracted their *POL* sequences. The *POL* sequences were used to construct a HMM profile of *POL*, which is highly conserved and has previously been used for *Gypsy* TE phylogenetic studies (7, 21, 49). The refined *POL* HMM profile was more generalizable across species and TE families compared with raw sequences and was used to identify TE insertions with high phylogenetic relatedness in each genome. Orthologs between the *D. melanogaster* consensus TEs and each species’ putative TEs were identified using reciprocal best hits. Briefly, we required each putative TE to be the best match for a *D. melanogaster* query TE and, conversely, for that *D. melanogaster* TE to be the best match for the putative TE sequence. Following this identification and assignment, downstream refining steps were applied to extend each hit into a full-length and high-quality consensus sequence (*SI Appendix*, Fig. S4). The pipeline outputs the consensus sequence of each available *D. melanogaster*-like *Gypsy*-family TE for each species (Fig. 2 *A*–*F*). When compared with a previous study (25), our framework captures a broader range of *D. melanogaster*-like TEs for most transposons tested (*SI Appendix*, Fig. S5).

In order to validate our pipeline, we sought to confirm that the sequences captured and annotated as *Gypsy*-family TEs were bona fide full-length elements rather than artifacts. LTR sequences provide a good estimate of this; the 5′ and 3′ LTRs of an LTR retrotransposon are identical at the time an insertion takes place, a by-product of the reverse transcription mechanism (50). Similar 5′ and 3′ LTR sequences therefore suggest a recent insertion. Here, we measured this by annotating the LTRs of the identified TE consensus sequences. We found that, for all TEs found in more than five species, 78% of LTR pairs in the consensus sequences shared >95% identity (*SI Appendix*, Fig. S6), suggesting that the TEs identified do, indeed, come from insertion events.

To validate our annotations further, we tested whether they are potentially controlled by the piRNA pathway, through mapping small RNA sequencing (sRNA-seq) data, where available, to our generated *Gypsy* TE libraries. As the piRNA pathway silences TEs by sequence complementarity, we expect piRNAs, captured by sRNA-seq, to map antisense to these sequences. By analyzing published sRNA-seq data (18, 51–57), we first confirmed that piRNAs sequenced in each species map to the TE library we constructed from that species (*SI Appendix*, Fig. S7, diagonal boxes). Next, we confirmed that piRNAs from the *melanogaster* clade and *Zaprionus* genus map among each other’s TEs (*SI Appendix*, Fig. S7), as would be expected for species with many similar TEs. Moreover, we showed that the piRNAs mapping to these TEs show the expected nucleotide frequencies (5′ U bias) (58), lengths (23 to 29 nt) and antisense mapping (*SI Appendix*, Fig. S8). In conclusion, piRNA sequencing data therefore aligns with the idea that our TE libraries largely consist of active or formerly active TEs, which are under the control of the piRNA pathway. Therefore, our pipeline provides a sensitive and accurate way of detecting high-fidelity *Gypsy* TE insertions and constructing consensus sequences of individual TE families in distantly related species.

### *Gypsy-*Family LTR Retrotransposons Are Widespread Across Drosophilids.

Having established a resource containing a total of 3,438 *Gypsy*-family consensus sequences across drosophilids, we were now able to study their evolutionary dynamics. Strikingly, conservation of *Gypsy*-family TEs varied widely across the phylogenetic tree, with anywhere between none or all (in *D. melanogaster*) of the 43 tested TEs—spanning five TE families (Fig. 2*B*)—found in individual species (at ≥70% similarity). *Gypsy/gypsy* (Fig. 2*A* and *SI Appendix*, Fig. S9*A*)*, Gypsy/mdg3* (Fig. 2*D* and *SI Appendix*, Fig. S9*A*), and *Gypsy/osvaldo* (Fig. 2*E* and *SI Appendix*, Fig. S9*A*) were found across the drosophilid phylogenetic tree, whereas *Gypsy/mdg1* (Fig. 2*C* and *SI Appendix*, Fig. S9*A*) and *Gypsy/chimpo* (Fig. 2*F* and *SI Appendix*, Fig. S9*A*) elements were not found in the most distantly related species. As expected, *D. melanogaster* TEs were most frequently found, and with highest similarity, in closely related species (*D. simulans-D. melanogaster*) (Fig. 2*G*). Moreover, the *Zaprionus* genus, known for extensive HTT with *D. melanogaster* (32, 33) shows high similarity between POL proteins for some *Gypsy* TEs (*ZAM, rover*) (Fig. 2*H* and *SI Appendix*, Fig. S9 *B* and *C*). Reassuringly, an alignment and resulting tree created from all *Gypsy* TE *POL* sequences shows that *POL* sequences annotated as the same *D. melanogaster Gypsy* clade cluster together (*SI Appendix*, Fig. S10*A*), reflecting their distinct evolutionary trajectories. Moreover, individual *POL* consensus sequences cluster together by TE and irrespective of species (*SI Appendix*, Fig. S10 *B*–*G*), consistent with a single origin of each TE. In conclusion, we detect widespread and conserved *Gypsy* TEs in a range of *Drosophila* species.

**Fig. 3.**
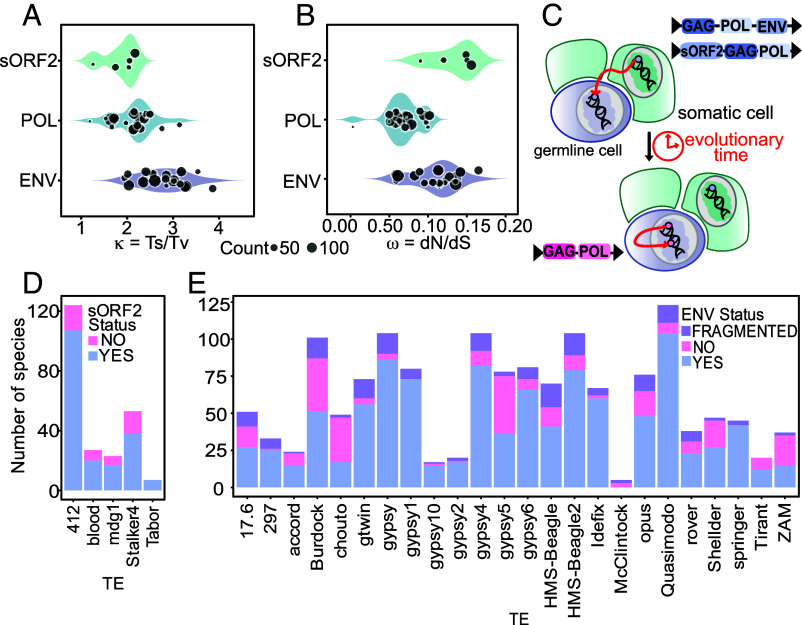
Analysis of *ENV* and *sORF2* indicates complementary functions and contrasting patterns of evolution. (*A*) Violin plots show the distribution of *Ts*/*Tv* values for ORFs in *Gypsy*-family TEs. (*B*) Shown is the same as A, but *dN/dS* values are displayed. (*C*) A schematic diagram shows the current model for *ENV* loss. TEs are initially active in the soma and require specialized ORFs to infect the germline. Upon a shift from somatic to germline expression, the ORF becomes redundant and, through natural drift, the ORF becomes lost or inactive. (*D*) A bar plot shows the status of the *sORF2* ORF in TE consensus sequences by number of species. (*E*) A bar plot shows the status of the *ENV* ORF in TE consensus sequences by number of species. *ENV* “presence” was considered to be an identified *ENV* equal to or longer than 900 nucleotides, while “fragmented” *ENV* was an *ENV* detected shorter than 900 nucleotides.

The *POL* sequence similarity between our annotated *Gypsy* TEs in species and their counterparts in *D. melanogaster* varied between 70% (the cutoff of our pipeline) and 100% similarity (Fig. 2 *A*–*H* and *SI Appendix*, Fig. S9). This suggests that TE ORFs may evolve between species. To better study the selective pressures and mutational biases that shape the evolutionary dynamics of the *Gypsy*-family TEs in drosophilids, we calculated the nonsynonymous to synonymous mutation (*dN/dS*) as well as the transition to transversion (*Ts/Tv)* ratios of our annotated *POL* sequences. The *dN/dS* and *Ts/Tv* scores show a high degree of purifying selection of the ORFs, with all *dN/dS* scores below 1 (Fig. 3 *A* and *B*). *POL* has *dN/dS* values on average 0.097 ± 0.005, in the same range as other *Drosophila* protein-coding genes (59). A likelihood ratio test (LRT) also favored the estimated selection models over neutral (*dN/dS* = 1) evolution (LRT statistic range: 71.7 to 34,282, *P* < 10^−15^ for all tests). The higher *dN/dS* and *Ts/Tv* values for *ENV* compared to other ORFs suggest that the *sORF2* and *ENV* ORFs may be undergoing purifying selection, like *POL*, albeit to a lesser degree (Fig. 3 *A* and *B*). These observations suggest that *Gypsy*-family elements in drosophilids are subject to purifying selection, that they remain functionally active, and are under evolutionary pressure to maintain the functionality of their coding sequences. There is, however, heterogeneity in these evolutionary constraints, implying that some ORFs are more strictly regulated than others.

### *Gypsy* TEs Show Distinct Insertion Preferences.

To better understand the dynamics of past TE invasions, we next examined the number and location of insertions for each TE subfamily. Many insertions were found to be highly similar to the consensus sequence (*SI Appendix*, Fig. S11), indicating that they may have recently invaded a species, while others were more diverged, suggesting older TE invasions which have allowed for the accumulation of mutations. For instance, *297* is likely younger in *D. melanogaster* (0.078% mean divergence across insertions) than in *D. mauritania* (2.2% mean divergence across insertions). Interestingly, the same TE can have different divergence profiles across species, indicating potentially differentially successful invasions. Similarly, several TEs have successfully invaded (i.e., have a large number of full-length copies) different species (*SI Appendix*, Fig. S12), and most species only have one such recently invading TE. Although TEs insert largely randomly in the genome, some TEs may have preferential insertion sites. Although TE divergence profiles varied across species (*SI Appendix*, Figs. S13 and S14), we did not observe a clear correlation between insertion location and TE divergence. Some TEs such as *blood* did, however, have more insertions in intragenic and promoter regions than others (*SI Appendix*, Fig. S15).

### Phylogenetic Incongruences Are Prominent in the Evolutionary History of *Gypsy*-Family TEs.

The evolutionary history of TEs is known to be complex and to contain many deviations from vertical inheritance, including HTT. Large incongruences between *POL* sequence phylogenetic trees and the species trees (*SI Appendix*, Fig. S16) highlight multiple putative instances of HTT in the evolutionary history of all *Gypsy/gypsy* TE families. To quantify all potential instances of HTT among the *Gypsy* TE phylogenetic tree, we identified species which had swapped “neighbors” between the species and TE trees. These phylogenetic incongruences exist both between closely related species, which may in part be due to incomplete lineage sorting or hybridization, and across clades, indicative of HTT, with multiple such incongruences observed between the melanogaster group and *Zaprionus* genus (*SI Appendix*, Fig. S17*A*), as recently reported (25). We speculate that the genomes with few *D. mel*-like TEs may be explained by limited opportunities for transposon exchange due to geographic segregation. The Hawaiian species (*D. silvestris*, *D. tanythryx*) are good examples of this as they consistently only share four TEs with *D. melanogaster* (*Circe, gypsy12, Quasimodo, HMS-Beagle2*) (*SI Appendix*, Fig. S9*A*). Indeed, TEs seem to have been passed to the Hawaiian species through a single putative HTT event (*SI Appendix*, Fig. S17*B*). Tropic niche segregation (60), for instance with cactophilic species (*D. mojavensis*), may contribute to the same phenomenon and has previously been shown to shape species’ HTT landscapes (61).

### *Gypsy/gypsy* and *Gypsy/mdg1* TEs Have Contrasting Patterns of Evolution.

To date, the *Gypsy/gypsy* and *Gypsy/mdg1* families are the only *Drosophila Gypsy* TEs known to contain proteins which enable cell-to-cell infectivity (7, 21) (Fig. 3*C*). Moreover, the ENV protein is ancestral to *Gypsy*/*gypsy* and has been lost across many *D. melanogaster* TEs (7). A change of expression of a TE from the soma to the germline would likely be accompanied by the gradual loss of the ENV protein due to random drift. To study this in detail, we focused on the conservation of *sORF2* and *ENV* across TE insertions in drosophilids. We asked whether full-length *ENV* and *sORF2* ORFs were consistently present or absent for a given *Gypsy/gypsy* or *Gypsy/mdg1* TE consensus sequence across all species which it has invaded.

The sORF2 protein was consistently present in ≥80% all *Gypsy/mdg1* TE consensus sequences (Fig. 3*D*). The consistently high prevalence of sORF2 in *Gypsy/mdg1* TEs, the presence of purifying selection (Fig. 3 *A* and *B*), combined with the broad prevalence of these TEs across species (most notably the TE *412*, which is found in 124 out of 249 species, suggests that *sORF2* is a near-universal component of *Gypsy/mdg1* TEs. In contrast, the ENV protein is not stably present or absent across *Gypsy/gypsy* TEs (Fig. 3*E*). Rather, it shows a high rate of *ENV* loss events (16 TEs with <80% *ENV* presence) throughout TE evolution. Moreover, *ENV* can also be detectable but fragmented and thereby nonfunctional, suggesting that it is no longer active but may have been in the past, as previously suggested in *D. melanogaster* (7). Interestingly, *ENV* presence/absence is not concordant with the species or TE trees (*SI Appendix*, Fig. S16), suggesting multiple, independent, gain or loss events across its evolutionary history. Notably, there were no co-occurrences of the sORF2 and ENV proteins, suggesting that they provide complementary and distinct mechanisms to enable a somatic niche for a TE.

### *Gypsy/gypsy* TE Expression Patterns Correlate With Specific TFBSs.

**Fig. 4.**
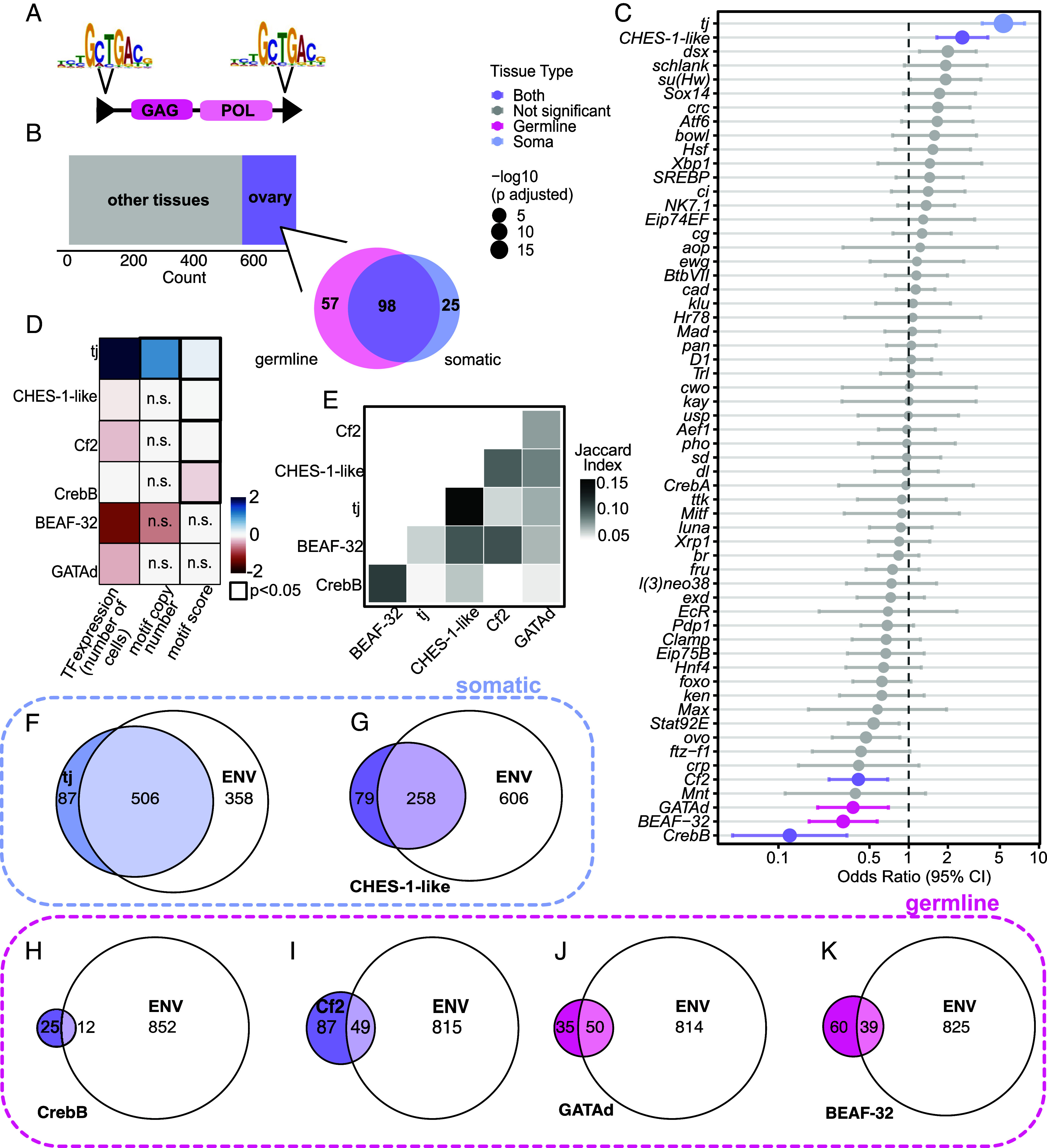
Specific TFBSs are associated with *ENV* presence or absence. (*A*) A schematic diagram shows the structure of an LTR retrotransposon and TFBS presence in the LTR regions. (*B*) A bar plot shows the number of ovary-expressed and non-ovary-expressed transcription factors in *D. melanogaster* and a Venn diagram shows the distribution of somatic, germline, and whole-ovary-expressed transcription factors in *D. melanogaster* ovaries. (*C*) The ORs for associations between TFBSs predicted in TE LTRs and *ENV* presence are displayed. The transcription factors shown are highly expressed in ovarian cells, defined as being detectable through scRNA-seq (64) in at least 20% of germline, somatic cells, or both. (*D*) A heatmap shows the log_2_ fold changes of the medians for the indicated metrics (proportion of cells with detectable expression, per-species number of TFBS motifs, or score of the motifs) for the six identified transcription factors between TEs containing full-length *ENV* and TEs which do not contain full-length *ENV*. The associated *P*-values, where significant, are the result of a Wilcoxon rank-sum test and are displayed in the heatmap. Crosses indicate nonsignificant ratios. (*E*) A heatmap shows the Jaccard index for TFBS co-occurrence in TE LTR regions for the six transcription factors with significant ORs. (*F*–*K*) Venn diagrams show the overlap between TEs containing full-length *ENV* and TEs containing the TFBS motif in their LTR regions, for the six transcription factors found to be significantly associated with *ENV* presence or absence: *tj* (*F*), *CHES-1-like* (*G*), *CrebB* (*H*), *Cf2* (*I*), *GATAd* (*J*), and *BEAF-32* (*K*).

A TE must contain all the regulatory elements required for its expression. The LTR regions of TEs contain small promoter regions that determine their transcription in specific tissues and cell types. They are known to contain TFBSs which determine TE expression patterns and levels (Fig. 4*A*). For instance, the transcription factor *traffic jam* (*tj*), in addition to its role in ovarian somatic cell development (62, 63), activates TEs as well as piRNA pathway components which, in turn, repress TEs (41, 42). To understand whether additional transcription factors may regulate transposon expression, we performed a computational screen for TFBS motifs in TEs in drosophilids. To focus the screen, we included only motifs for transcription factors expressed in the *D. melanogaster* ovary (Fig. 4*B*). We asked whether the presence or absence of motifs in the LTR region of a TE consensus sequence could be used to predict its *ENV* status. This relies on the hypothesis that *ENV*-containing TEs are expressed in the soma and TEs which do not contain functional *ENV* are expressed in the germline.

We used a total of 1,267 5′ LTR region sequences of which 864 were associated with a full-length *ENV* ORF. TE families where all species had the same *ENV* status (e.g., absent in all *McClintock* TEs) were excluded from this analysis. Using a mixed effects logistic regression with TE family modeled as a random effect, we identified that *tj* [Odds Ratio (OR) = 5.06, *P*_adj_ = 5 × 10^−20^] and *CHES-1-like* (OR = 2.78, *P*_adj_ = 8 × 10^−7^), both of which are somatically expressed, were significantly associated with *ENV* presence. Moreover, four transcription factors expressed in the germline were significantly associated with *ENV* absence: *Cf2* (OR = 0.323, *P*_adj_ = 4 × 10^−6^), *GATAd* (OR = 0.549, *P*_adj_ = 0.043)*, CrebB* (OR = 0.113, *P*_adj_ = 1 × 10^−5^), and *BEAF-32* (OR = 0.340, *P*_adj_ = 1 × 10^−4^) (Fig. 4*C*). Of note, while three of six identified transcription factors were detectible in both the germline and the soma (Fig. 4*C*, purple hits), they were in all cases most broadly detectible in the tissue where they were predicted to regulate TEs (Fig. 4*D*). Comparing TFBS motifs across TEs with our without *ENV*, we found that motif scores were, for *tj*, *CHES-1-like*, *Cf2* and *CrebB*, significantly stronger in the TEs they were predicted to regulate (Fig. 4*D* and *SI Appendix*, Fig. S18), and *tj* contained significantly more TFBSs in the LTRs of TEs without an ENV protein (Fig. 4*D* and *SI Appendix*, Fig. S19). The predictors of somatic expression, *tj* and *CHES-1-like*, were also the most likely to co-occur as the pair had the highest Jaccard index (Fig. 4*E*). *tj*’s binding motif was the most abundant and present in at least one species for nearly all the *Gypsy/gypsy* TEs tested (Fig. 4*F*). Moreover, TFBSs associated with *ENV* presence were primarily found in TEs which contain *ENV* (Fig. 4 *F* and *G*) and, conversely, ones associated with *ENV* absence were primarily found in TEs which lack the ORF (Fig. 4 *H*–*K*). Finally, conservation analysis of our most significant hits confirmed that they are present and highly conserved across drosophilid species, further indicating their potentially conserved role in determining TE expression patterns (*SI Appendix*, Fig. S20).

## Discussion

Here, we took advantage of recent efforts in long-read whole-genome sequencing across drosophilids (43, 44) to study TEs at a large scale and across multiple species. Previously, similar genome assemblies allowed the identification of piRNA clusters in multiple non–*D. melanogaster Drosophila* species, yielding insights into the regulation of TEs (18), as well as enabling evolutionary insights into other repetitive genomic elements (65). High-quality genome assemblies have also enabled recent in-depth studies of coevolution of TEs with their host, such as in *D. virilis* (66). Here, we instead used the available drosophilid genomes to construct *Gypsy*-family TE libraries for 249 species. The resulting resource allowed extensive evolutionary studies on TEs across multiple species spanning 50 My of evolution.

Our results highlight the widespread nature of TE transfer, as putative HTT events were observed in the evolutionary history of all *Gypsy*-family TEs studied. In line with similar recent findings in other organisms, this establishes TE mobility, including HTT and introgression as key drivers in shaping the mobile genome (67) and particularly in *Drosophila* (68, 69). In drosophilids, recent work has characterized potential HTT events among species and suggested that HTT events occur when species are in close geographical proximity (25). Currently, the vast number of phylogenetic incongruences between TE trees and host species trees make it difficult to pinpoint an exact donor and acceptor of putative HTT events. While clustering and topological incongruence between the TE and species phylogenetic trees is a good indicator of HTT (70, 71), several alternative methods exist to identify HTT events. These include pairwise comparisons of evolutionary divergence of all shared genes followed by comparing the TE divergences against a baseline determined by those of BUSCO genes (25). Moreover, the vertical hybridization and inference of congruence analysis (VHICA) method compares signatures of selection (*dS*) between genes of interest and a user-determined baseline of conserved, vertically transferred genes (72). Despite this, HTT is difficult to differentiate from other phenomena such as recombination and coalescence. We hope that our TE annotations combined with larger-scale genomic surveys and future tree reconciliation methods will enable this.

TE-encoded proteins of viral origin that enable cell-to-cell movement of the elements are of interest due to their ability to enable a TE to infect the germline (7, 21). Previous work has indicated that insertions of the same TE, *rover*, in *D. melanogaster* can encode both active and inactive protein variants, a distinction reflected in the insertions’ expression patterns (7). On a genus-wide level, our data show that *Gypsy/mdg1* TEs have a highly conserved sORF2 protein, present in nearly all detected consensus sequences across drosophilids, whereas *Gypsy/gypsy* TEs have lost the ENV protein repeatedly throughout their evolutionary histories. A central premise guiding our analyses is the well-supported dichotomy in *D. melanogaster* that TEs containing *ENV* or *sORF2* are expressed in the soma whereas elements lacking a functional copy of either of these ORFs reside in the germline (7, 21). Although we did not directly assay tissue-specific expression for the drosophilid species analyzed in this study, we consider this assumption highly robust due to the extensive evidence in *D. melanogaster* and known role of ENV and FAST-like proteins in mediating cell-to-cell transfer. Consistent with its evolutionary plasticity, *ENV* has a higher, on average, *dN*/*dS* ratio relative to the other proteins and independently of the species or TE phylogenies.

Transcription of TEs is mediated by TFBSs in their LTR regions (41, 42, 73). In *Gypsy/gypsy* TEs, we identified two transcription factors significantly associated with somatic expression, *tj* and *CHES-1-like,* and four significantly associated with germline expression: *Cf2*, *GATAd, CrebB,* and *BEAF-32*. These factors were previously characterized as crucial in *D. melanogaster* gonad development (62, 63, 74–76). Interestingly, *BEAF-32* also regulates the non-LTR TEs and the piRNA pathway (77). Hijacking key developmental pathways likely means that the transcription factors required for TE expression will be present following a jump to another species. Another benefit is likely that the host is unable to render a key transcription factor dysfunctional to halt a TE invasion. Some transcription factors whose TFBS motifs were not significant hits in our screen may still be functional in activating TE expression and their redundancy may serve as a fail-safe mechanism to ensure transcription of the TE insertion across many cellular niches.

In conclusion, we present a resource containing *D. melanogaster* TE consensus sequences in 249 high-quality drosophilid genomes. We highlight the distinct evolutionary mechanisms by which some ORFs evolve and shed light on the highly conserved mechanisms TEs use to ensure their transcription in their host genomes. Our results and dataset will enable studies of TEs coevolution with the host genome in the future, particularly focusing on TE transcription and cell type–specific expression.

## Materials and Methods

### Genome Assemblies.

We used previously published resources of long-read assemblies (43, 44, 78) as listed in Dataset S3.

### Genome Assembly Quality Control.

Genome quality control was carried out using calN50 (https://github.com/lh3/calN50), checking the number of sequences (NN) and genome contiguity (N50). A genome assembly was considered to have passed the quality threshold if N50 > 1,000,000 and NN < 3,000. For species with more than one available genome assembly, the highest quality assembly was kept.

### De Novo TE Annotations.

Initial de novo TE libraries were built for each genome using RepeatModeler (v2.0.7) (79) *BuildDatabase* followed by *RepeatModeler* (-threads 4 -LTRStruct). These were inputted as the RepeatModeler library (--rmlib) into EDTA (v2.2.2) (47) (--species others --step all --anno 1 --threads 4 --force 1). SINE elements were not included in this analysis due to low genomic coverage, likely due to difficulties being captured by the automated tools used (5, 6). The percentages of the genome covered by each TE class, superfamily, and family were extracted from the RepeatMasker *.tbl output file generated by EDTA. To quantify how much of the *Gypsy*-family coverage that was *D. mel*-like, we compared the total *Gypsy*-family genomic coverage to that of the *D. melanogaster* reference transposon library estimated using RepeatMasker (48). To compare the genomic proportions of TE groups, a Wilcoxon signed-rank test was utilized. We opted for a paired nonparametric test as the percentages compared are paired by host species. We used the wilcox.test function from the stats (v4.5.1) package in R (v4.5.1) with the paired = TRUE flag.

### Phylogenetic Tree Visualization.

A previously generated phylogenetic tree for 704 drosophilidae species (80) was used to display our species onto a phylogenetic tree. The phylogenetic tree was imported using treeio (v1.28.0) (81) and species not included in our study were removed. The tree and metadata were visualized using ggtree (v3.12.0) (82) and ggtreeExtra (v1.19.1) (83).

### ORF Identification Using HMMs.

TE libraries (18) from 193 previously studied *Drosophila* genome assemblies were filtered to only include *Gypsy*-family LTR retrotransposons. Their *POL* sequences were extracted by mapping all consensus sequences to RepeatPeps.lib in RepeatMasker (v4.1.2) (48) using blastx (v2.16.0) (84). *GAG* and *ENV* ORFs were extracted in the same way. The resulting ORF nucleotide sequences were assembled into a HMM using hmmer’s (v3.4) hmmbuild (85–87) using Henikoff position-based weights. A HMM for *sORF2* was constructed using available *sORF2* sequences (21). The *POL* HMM was used to identify *POL* sequences in all drosophilid genomes using nhmmer with default parameters. True hits were considered to be ones with an e-value <10^−20^ and length >2,500 nucleotides. The HMMs developed are available on https://github.com/annamaria23/retrotransposon-atlas/tree/main/2_ORF_HMMs.

### TE Annotation.

The *D. melanogaster* reference *Gypsy* TE database was built using a preexisting, manually semicurated, TE database (https://github.com/bergmanlab/drosophila-transposons) to which we manually added eight recently identified *Gypsy*-family TEs: *chouto, Chimpo, Bica* (7)*, MLE, Souslik, Transib1* (31)*, Spoink and Shellder* (69). In cases where *POL* was not already annotated, it was identified using the same HMM method as for putative TEs (cf. *ORF identification using HMMs*) or using blastx (v2.16.0, default parameters) against *POL* sequences in RepeatPeps.lib (RepeatMasker, v4.1.2) and the longest sequence was selected and manually validated. MMseqs2 (v18.8cc5c) (88) *createdb* followed by *rbh* and *convertalis* with default parameters were used to identify reciprocal best hits between all putative *POL* sequences in a genome and the *POL* sequences of the curated *D. melanogaster* TEs.

### Consensus Sequence Creation.

To create full-length TE consensus sequences, the annotated *POL* ORFs were mapped back to each genome using blastn (v2.16.0) (84) filtering for 90% identity, 1e-20 E value cutoff, and for the resulting hits to be longer than half that of the query sequence. The top 20 sequences were selected, based on E value. The resulting sequences were extended 5,000 nucleotides up- and down-stream using bedtools slop (v2.31.1) (89) In cases where three or more sequences were present, a multiple sequence alignment (MSA) was constructed using mafft (v7.526, --reorder --auto) (90, 91). CIAlign (v1.1.4) (92) was used to refine the MSA by filtering divergent sequences (--remove_divergent --remove_divergent_minperc 0.3 --remove_short) and low-coverage regions (--crop_divergent --crop_divergent_min_prop_nongap 0.8 --crop_divergent_min_prop_ident 0.8 --crop_ends --remove_insertions --insertion_max_size 3000) and generate a consensus sequence of the TE (--make_consensus). The full consensus sequence of the putative TE was mapped back to the genome using blastn (v2.16.0) (84) with default parameters and the 5′ and 3′ ends of the TE sequences were trimmed to remove nucleotides with low mapping coverage to the genome using a custom Perl (v5.32.1) script (crop_zero_coverage.pl from https://github.com/susbo/Drosophila_unistrand_clusters) (93). In cases where fewer than three *POL* sequences were identified in the genome from the initial blast step (this would represent TEs with only one or two insertions in the genome), the one with the lowest E value was selected and considered the consensus sequence.

### Annotation of TE Consensus Sequences.

To obtain the LTR regions of putative TEs, blastn (v2.16.0) was used on the sequence against itself. The longest identified sequences where the 5′ LTR ended before the start codon of the *GAG* ORF and the 3′ LTR began after the stop codon of the *POL* ORF were considered to be the LTR regions. TE sequences were trimmed such that they began at the first nucleotide of the 5′ LTR and ended at the last nucleotide of the 3′ LTR.

### TE Annotation Benchmarking.

We compared the annotations of this work with those of a recent study (25) found on https://github.com/rpianezza/Drosophilids-TE-biogeography/blob/main/D.melanogaster/data/TEs.clean.score. We used the provided metadata file (te-metadata.csv) to convert the TE names and filtered for a TE score ≥0.5, following the previously published methodology. Lastly, we filtered for *Gypsy-*family elements and only included TEs and species for our downstream analysis that were included in both studies.

### Similarity Between LTR Sequences.

blastn (v2.16.0, default parameters) between the 5′ and 3′ LTRs of TE consensus sequences was used to obtain their pairwise similarity.

### Publicly Available sRNA-Seq Data.

We used publicly available sRNA-seq data from previous studies (51–56), which were also used in (18), to which we added sRNA-seq data from *D. triauraria* and five *Zaprionus* species (57). An overview of the libraries used is available in Dataset S2.

### sRNA-Seq.

All sRNA-seq data were processed using the same analysis pipeline as (18). Briefly, after removing an abundant rRNA, sequencing adapters, any flanking random nucleotides and reads mapping to miRNAs, as described (18), the remaining reads were mapped to the TE consensus sequences using bowtie (v1.2.3, -S -n 2 -M 1 -p 20 --best --strata --nomaqround --chunkmbs 1024).

### *dN*/*dS* and *Ts*/*Tv* Scores.

The HMM-based consensus ORF sequence was refined and then translated using EMBOSS’ *getorf* (v6.6.0.0, -find 3 -minsize 900) (94) and *transeq* functions. After duplicate sequences were removed using seqkit rmdup, amino acid MSAs were obtained using mafft (v7.526) (90, 91) with default parameters. The amino acid MSA and sequence files were used to generate a codon alignment using PAL2NAL (v14) (95) and phylogenetic trees were generated from this MSA using IQTREE2 (v2.3.6) (96) with the -m MFP flag. *dN/dS* and *Ts/Tv* scores were obtained using PAML’s *codeml* (v4.10.7, CodonFreq2; model 0; NSsites 0; fix_omega 0; omega 1) (97) function. The neutral evolution model (M(0) = 0, fix_omega 1) was compared to the selection model (M(0) = 1, fix_omega 0) using a LRT [2* (lnL_M1_ − lnL_M0_)] for each TE ORF consensus sequence individually. The resulting LRT statistic was compared against a χ^2^ distribution with one degree of freedom.

### Identification of Individual TE Insertions and Proximity to Protein-Coding Genes.

TE libraries for each genome were searched in the species’ genome using RepeatMasker (48), specifying the library with the -lib flag, BUSCO (v6.6.0) for the *drosophilidae_odb12* lineage with default parameters was used to identify protein-coding genes in each genome. Lastly, bedtools (v2.31.1) (89) *closest* was used to identify the closest protein-coding gene to each TE insertion.

### Phylogenetic Trees from *POL* TE Consensus Sequences.

MSAs of *POL* nucleotide sequences were generated using mafft (v7.526) (90, 91) with default parameters and refined using CIAlign (92) (v1.1.4, --crop_divergent --crop_divergent_min_prop_nongap 0.8 --crop_divergent_min_prop_ident 0.8 --remove_divergent --remove_divergent_minperc 0.3 --crop_ends --remove_insertions --insertion_max_size 3000 --remove_short). For individual TE *POL* ORFs, phylogenetic trees were constructed from the resulting MSAs using IQTREE2 (v2.3.6) (96), selecting the best substitution model using ModelFinderPlus (-m MFP). The tree search was initiated from a pool of 100 initial parsimony trees (--ninit 100). The reliability of the internal branches was tested using the SH approximate LRT (-alrt 1000), approximate Bayes test (-abayes), and ultrafast bootstrap (-B 1000) with an additional optimization step using Nearest-Neighbor Interchange applied to each bootstrap replicate (-bnni). A fixed random seed (--seed 42) was used. When computing phylogenetic trees for all TE ORFs, FastTree (v2.2.0) (98) with default parameters and removed identical sequences.

### Identifying Putative HTT Events.

Putative HTT events were called by comparing the phylogenetic topologies of the TE tree and species tree using the ete3 (v3.1.3) (99) package in Python (v3.10.8). For each species, its closest relatives are defined in both the species tree and the TE tree as the descendant leaves with the same parent node. For each species, if its set of “neighbor” species in the gene and species trees do not share at least one element, HTT is considered to have taken place between the target species and the “new” relative on the TE tree. To ensure reliability, only nodes with an ultrafast bootstrap support value above 70% were considered.

### Distance Between *ENV*-containing TEs in Phylogenetic Trees.

The *cophenetic()* function was used in R (v4.5.1) to calculate distances between selected nodes in trees. The mean value of the lower triangle of these distances was then taken.

### Motif Discovery.

Motifs in LTR regions were identified from the *D. melanogaster* JASPAR TFBS motifs (100) using FIMO (v5.5.5, default parameters) (101). The TFs were filtered for somatic, germline, or whole-ovary expression based on processed, publicly available single-cell RNA-seq data (102) accessed on https://wiki.flybase.org/wiki/FlyBase:Downloads_Overview.

### Screen for Transcription Factors that Regulate TE Niche Expression Using Logistic Regression.

We predicted the *ENV* status based on the presence/absence of TFBS motifs using a generalized linear mixed effect model (GLMM) with a binomial error distribution and a logit link function with the *glmer()* function from the lme4 (v1.1-37) (103) in R (v4.5.1). Presence of a TFBS motif was treated as a fixed effect and TE identity was included as a random intercept. The bound optimization by quadratic approximation (bobyqa) optimizer was used with the iteration limit increased to 100,000. Raw *P*-values were adjusted to control for the false discovery rate using the Benjamini–Hochberg procedure.

### TFBS Co-Occurrence Analysis.

The co-occurrence of TFBS in LTR regions of TEs was computed in R (v4.5.1) using the Jaccard Index, defined as:

$$J\left(TF_{1},\;TF_{2}\right)=\frac{TF_{1}\cap TF_{2}}{TF_{1}\cup TF_{2}}.$$

### Mapping *D. melanogaster* Transcription Factors to Drosophilid Reference Genomes.

To identify transcription factors of interest in the 249 fly genomes, the longest isoform from *D. melanogaster* was obtained from FlyBase (https://flybase.org/) and mapped to the genomes using miniprot (v0.18-r281) (104) with the -I flag to automatically select the maximum intron size based on genome length and -gtf. gffread (v0.12.7) (105) was then used to obtain the coding (-w) and protein (-y) sequences. Finally, search36 (v36.3.8 g) (105) was used to obtain the SW score between the *D. melanogaster* protein sequences and the ones identified in other species. The highest SW score was kept for each species in cases where multiple hits were found. Similarity values <50% were discarded and the final SW scores were normalized to the length of the corresponding protein in *D. melanogaster*.

## Supplementary Material

Appendix 01 (PDF)

Dataset S01 (XLSX)

Dataset S02 (XLSX)

Dataset S03 (XLSX)

## Data Availability

The scripts required to implement our transposon annotation pipeline, together with our curated transposon consensus sequences and their metadata are available on GitHub (https://github.com/annamaria23/retrotransposon-atlas) and have been deposited in Zenodo (https://doi.org/10.5281/zenodo.20395944) (106). Previously published data were used for this work. All genome assemblies used in this study are publicly available (43, 44, 78).
